# CANDLE Syndrome As a Paradigm of Proteasome-Related Autoinflammation

**DOI:** 10.3389/fimmu.2017.00927

**Published:** 2017-08-09

**Authors:** Antonio Torrelo

**Affiliations:** ^1^Department of Dermatology, Hospital Infantil del Niño Jesús, Madrid, Spain

**Keywords:** CANDLE, neutrophilic dermatosis, proteasome, immunoproteasome, interferonopathy, autoinflammation

## Abstract

CANDLE syndrome (*C*hronic *A*typical *N*eutrophilic *D*ermatosis with *L*ipodystrophy and *E*levated temperature) is a rare, genetic autoinflammatory disease due to abnormal functioning of the multicatalytic system proteasome–immunoproteasome. Several recessive mutations in different protein subunits of this system, located in one single subunit (monogenic, homozygous, or compound heterozygous) or in two different ones (digenic and compound heterozygous), cause variable defects in catalytic activity of the proteasome–immunoproteasome. The final result is a sustained production of type 1 interferons (IFNs) that can be very much increased by banal triggers such as cold, stress, or viral infections. Patients start very early in infancy with recurrent or even daily fevers, characteristic skin lesions, wasting, and a typical fat loss, all conferring the patients a unique and unmistakable phenotype. So far, no treatment has been effective for the treatment of CANDLE syndrome; the JAK inhibitor baricitinib seems to be partially helpful. In this article, a review in depth all the pathophysiological, clinical, and laboratory features of CANDLE syndrome is provided.

## Definition

CANDLE is an acronym standing for *C*hronic *A*typical *N*eutrophilic *D*ermatosis with *L*ipodystrophy and *E*levated temperature (1–3). CANDLE syndrome is an autoinflammatory disease (AID) characterized by the appearance of recurrent fever in the first months of life, along with characteristic skin lesions, lipodystrophy, and manifestations of multisystem inflammation. Mutations in different genes encoding protein subunits of the proteasome–immunoproteasome system are the cause of CANDLE syndrome.

## History

CANDLE syndrome was described in 2010 by Torrelo et al. (1). They reported on four children collected in two centers in Madrid and Chicago, two of whom were siblings, who showed striking skin lesions which on histopathology displayed an infiltration of immature, myeloid, mononuclear cells, resembling leukemia cutis. Because in many parts of the skin biopsies there was some maturation into polymorphonuclears and karyorrhexis, a type of yet undescribed “neutrophilic dermatosis” was suspected. The skin lesions had appeared very early in infancy, in attacks that occurred after common triggers (especially cold and viral infections), but some degree of skin involvement was present all the time. Furthermore, the patients had recurrent, almost daily, fevers or temperature elevations below 38.3°C, also since very early in life. The disease seemed to cause some general growth delay, and after more than 10 years of follow-up most patients looked wasted, with a striking loss of fat. With all these features, the chronic eruption with skin neutrophilic and mononuclear immature infiltration, the fevers and the lipodystrophy, an acronym was coined. The dermatological aspects were emphasized in this description, because they had been the most constant features, but it was recorded that the patients had suffered unexplained inflammatory attacks in many different organs of the body, such as the central nervous system (CNS), cartilage, joints, testes, and many others. One of the patients died of an attack of “carditis,” but autopsy was not performed.

Also, in 2010, Garg et al. (4) reported three adult patients with a disease they named JMP (*J*oint Contractures, *M*uscle atrophy, microcytic anemia, and *P*anniculitis-induced lipodystrophy syndrome). The authors emphasized the lipodystrophic features that were related to panniculitis, and the joint features, but did not mention on the skin manifestations of the disease. However, they anticipated that their patients would suffer a disease of the innate immune system.

A similar constellation of signs had been reported in the Japanese literature. It had been reported by Nakajo in 1939 and Nishimura in 1950, under the names “secondary hypertrophic osteoperiostosis with pernio,” a syndrome with nodular erythema, elongated and thickened fingers, and emaciation, and “hereditary lipomuscular atrophy with joint contracture, skin eruptions and hyper-γ-globulinemia” (5). An eponym for the syndrome was proposed by the Japanese authors as “Nakajo–Nishimura syndrome.” Overall, patients described started in early infancy with a pernio-like rash, periodic high fever, nodular erythema-like eruptions, and myositis. Lipoatrophy and joint contractures occurred progressively in life, mainly on the upper part of the body, leading to a very characteristic facial appearance.

After the description of CANDLE and JMP, and the appearance of new cases from Japan, a number of cases from different parts of the world were published. It was later disclosed that most patients described under these descriptions had homozygous or compound heterozygous mutations in the gene *PSMB8*, encoding the subunit β5i of the immunoproteasome (2, 6–8). However, some patients with CANDLE syndrome did not bear mutations in *PSMB8* (2). Further genetic studies disclosed that some patients have homozygous and compound heterozygous mutations in other subunits of the proteasome–immunoproteasome, as well as digenic heterozygous mutations in two different genes encoding subunits (9). Finally, mutations in the proteasome maturation protein (*POMP*) gene have also been reported in a patient of Lebanese origin that had been reported by Mégarbané et al. (9, 10) in 2002 under the name “unknown autoinflammatory syndrome associated with short stature and dysmorphic features.”

Several terms have been proposed to encompass the three denominations, such as PRAAS (proteasome-associated autoinflammatory syndrome) (11) or ALDD (autoinflammation, lipodystrophy, and dermatitis) (12). However, all of them represent the same entity, and it seems that the most accepted nomenclature is CANDLE syndrome. The general appearance of consumption in the patients, like a burntout candle, emphasizes the name of the syndrome.

## Pathophysiology

Overall, proteasome–immunoproteasome dysfuntion causes a continuous state of inflammation with exacerbations in CANDLE syndrome. Proteasome–immunoproteasome dysfunction leads to constitutional hypersecretion of type 1 Interferons (IFNs), which by several mechanisms will lead to accumulation of waste proteins within the cells (13). Thus, further proteasome–immunoproteasome activity is required, which cannot be achieved. Accumulation of waste proteins within the cell causes cellular stress, which in turn stimulates type 1 IFN, and finally closes the circle of inflammation. Common triggers, such as cold, physical or psychical stress, banal infections or others cause stimulation of type 1 IFN secretion, and thus provoke severe inflammatory attacks that can occur in any organ of the body.

### The Proteasome–Immunoproteasome System

**Figure 1:**
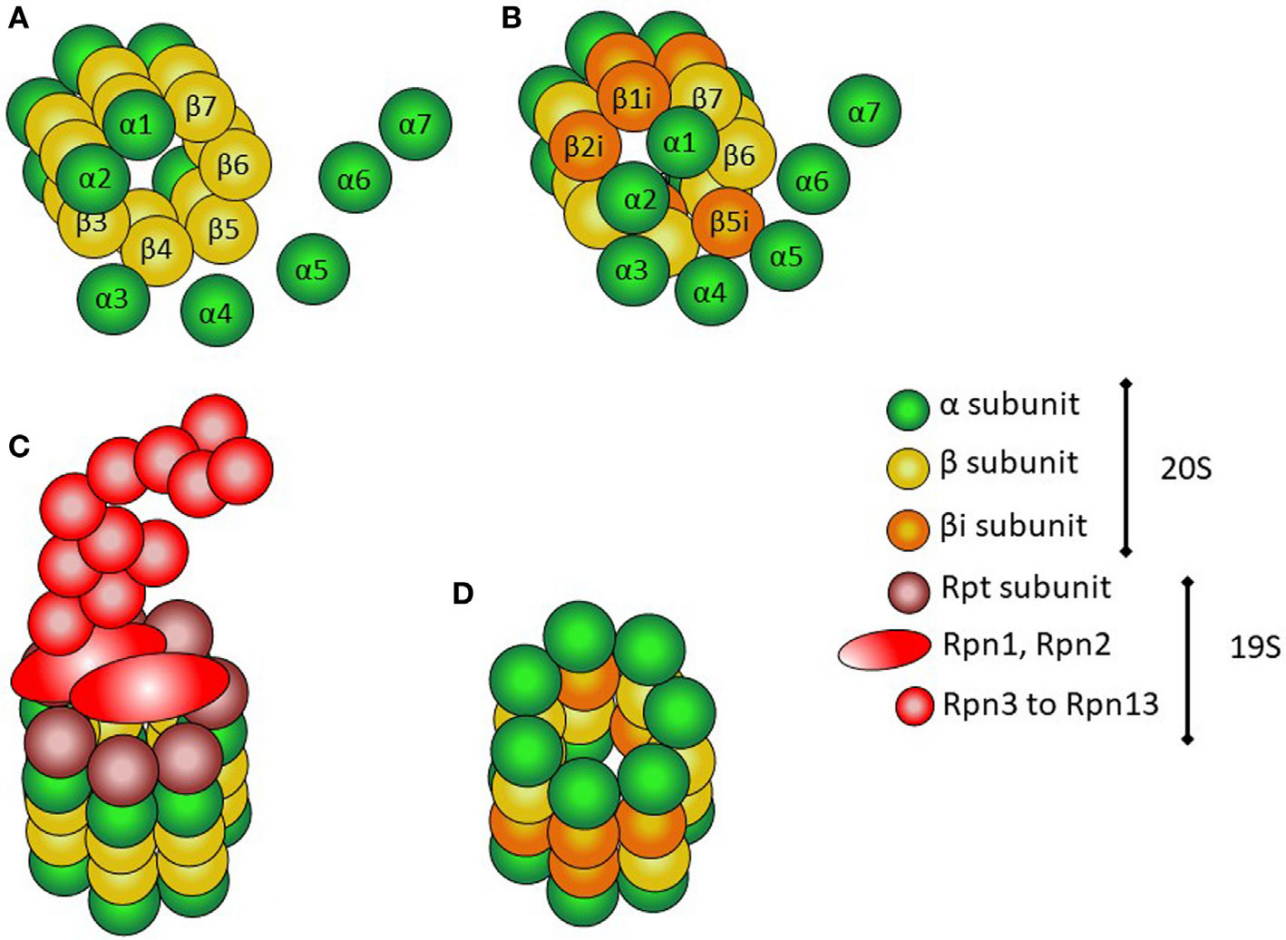
Structure of proteasomes. **(A)** Assembly of α and β subunits to form the 20S (core) complex of the constitutive protesome. **(B)** Assembly of the inducible β subunits to form the 20S (core) of the immunoproteasome. **(C)** 26S proteasome with the 20S core particle and the 19S regulatory complex. **(D)** 20S (core) immunoproteasome.

The proteasome is a multiprotein structure present both in the nucleus and the cytoplasm of all eukaryotic cells (Figure 1) (14). It is constitutively expressed, and has multicatalytic activity. It has a cylindrical shape, and there are several types. All of them contain at least two different complexes: the 20S or core complex, which contains the proteolytic activity, and the 19S or regulatory complex, responsible for recognizing ubiquitinated proteins and transporting them into the 20S complex. The 20S complex is composed of two α-rings flanking two β-rings; each ring is composed of seven different protein subunits, named α-1 to α-7 and β-1 to β-7.

The immunoproteasome is very similar to the constitutive proteasome (Figure 1). The 19S complex of the proteasome is replaced with the PA289 (or 9S) complex, and the β1, β2, and β5 subunits in the beta rings are substituted by specialized units, named β1i, β2i, and β5i (i = inducible) (14). The β1 subunits have caspase-like activity, β2 subunits have trypsin-like activity, and β5 subunits have chemotrypsin-like activity (9). Immunoproteasome formation is mainly induced by IFNs, to account for an increased demand of catalytic activity within the cell.

The assembly and maturation of proteasome and immunoproteasomes are governed by facilitating proteins. POMP is essential for proteasome formation and is strongly involved in the incorporation of the β5i subunit into the immunoproteasome. Proteasomal β subunits but β3 and β4, are synthesized as preforms that require autocatalytic cleavage during assembly to liberate the active-site threonines (9, 15, 16).

The proteasome is mostly involved in the removal of waste intracellular proteins. The immunoproteasome is also responsible for the degradation of foreign proteins. Whereas the proteasome is constitutively expressed in every cell, only proinflammatory cytokines and mostly type 1 IFNs induce the immunoproteasome formation. However, immunoproteasomes are constitutively expressed in hematopoietic cells (17, 18). The catalytic activity of the proteasome and the immunoproteasome cleaves protein substrates to generate smaller peptides that can be easily removed from the cell or may act as antigens that can be presented through the MHC type I molecules to the adaptive immune system. In this way, more specifically the immunoproteasome acts as a link between innate and adaptive immunity.

### Normal Functioning of the Proteasome–Immunoproteasome System

**Figure 2:**
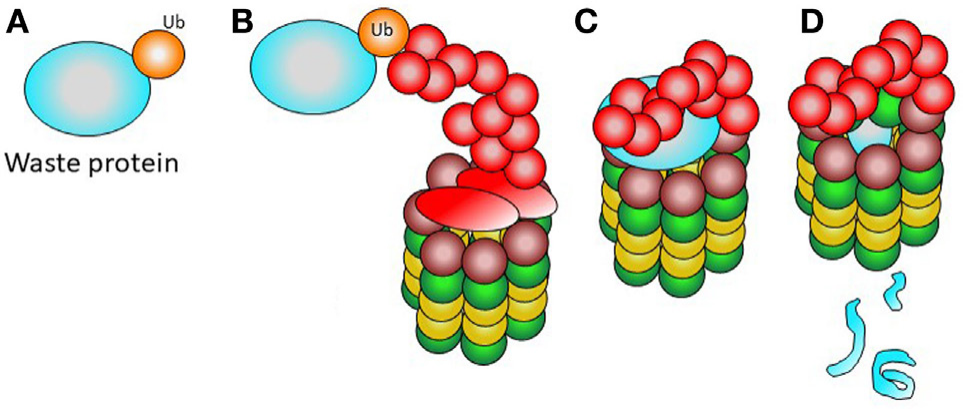
Normal function of the proteasome. **(A)** A waste protein fated to elimination is marked with ubiquitin. **(B)** The 19S complex recognizes the ubiquitinated protein. **(C)** The ubiquitinated waste protein is entered into the proteasome. **(D)** The catalytic activity of the 20S complex renders small products, easy to remove from the cell.

Cellular proteins destined for degradation or cleavage should be first marked with ubiquitin. Ubiquitinization allows for recognition by the 19S complex. The ubiquitinized protein is entered into the 20S particle, which degrades the protein through enzymatic proteolysis (Figure 2). The small peptides resulting from degradation can thus be easily removed or enter the endoplasmic reticulum to be eventually presented to T lymphocytes by means of the MCH type I on the cell surface (19, 20).

**Figure 3:**
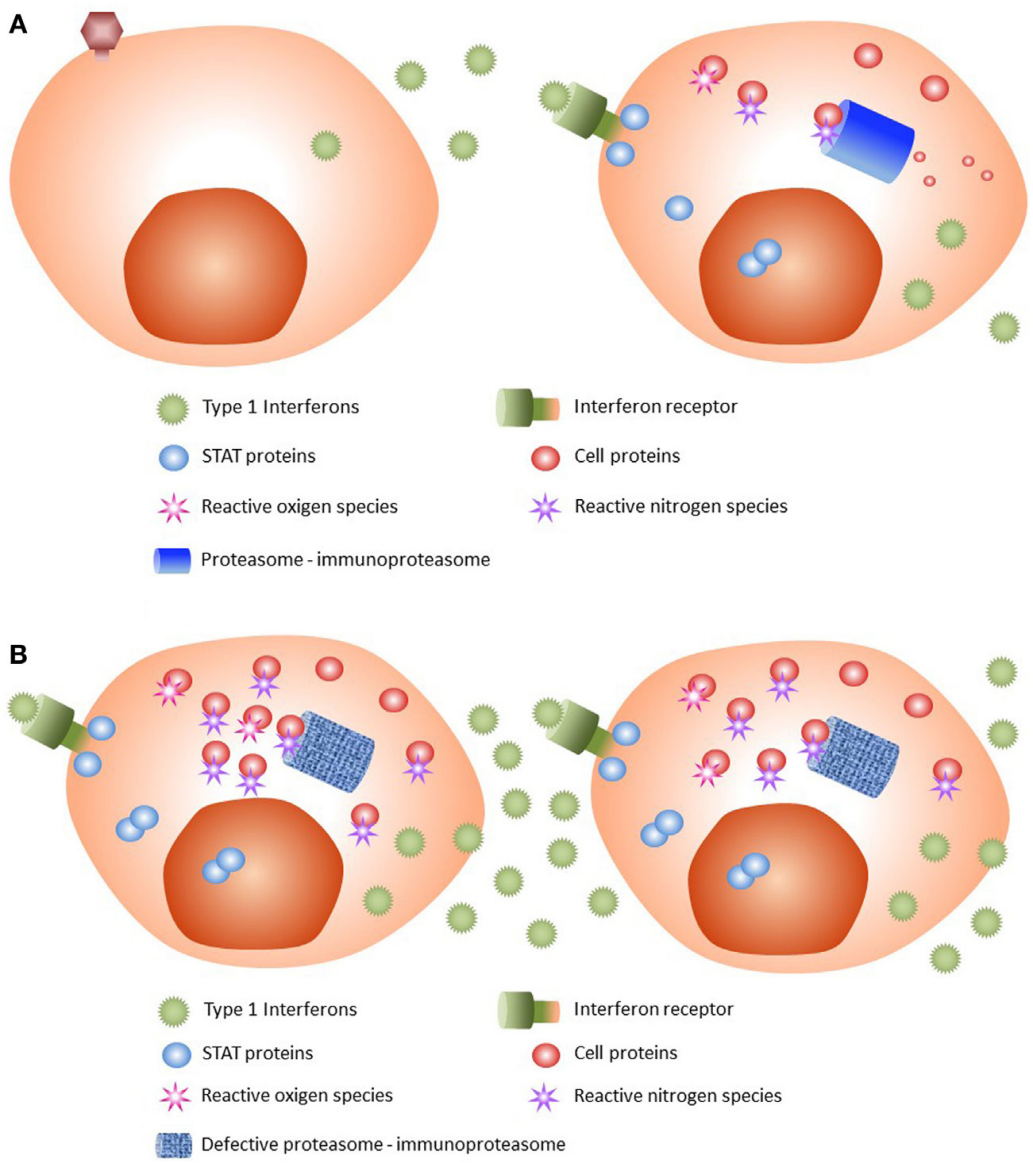
Pathophysiology of CANDLE syndrome. **(A)** Normal state. After viral infection, type 1 interferons (IFNs) are released by the infected cell. IFNs are recognized by their receptor, with activation of JAK/STAT pathway and subsequent dimerization of STAT proteins. The STAT dimers enter the nucleus and stimulate transcription of type 1 IFNs. JAK/STAT activation produces reactive oxygen and nitrogen species that are damaging to cell proteins. These damaged proteins, and others generated by cell catabolism, are removed by the proteasome and the immunoproteasome; the latter is stimulated by type 1 IFNs. **(B)** CANDLE syndrome. After IFN activation, cells with mutated proteasome–immunoproteasome will not be able to remove all waste proteins, which will accumulate and be poly-ubiquitinized. As a result, cellular stress occurs, leading to increased type 1 IFNs production. The high levels of secreted type I IFNs recruit inflammatory cells that will cause tissue damage.

Viral infections or other triggers such as cellular stress or cold can induce secretion of type 1 (α or β) IFNs. When IFNs are recognized by their cell surface receptor, transphosphorylation of JAK1 and TYK2 occurs (21), as the first step of JAK/STAT signaling pathway activation. As a result of activated JAKs, the STAT proteins STAT1 and STAT2 dimerize and enter the nucleus, leading to transcription of type 1 IFN genes (Figure 3A). On the other hand, danger signals such as an irritant or infection provoke inflammation, which is associated with the production by immune cells of microbiocidal reactive oxygen and reactive nitrogen species during the innate immune response (22). Such increased oxidative stress conditions have profound consequences for the functional integrity of proteins.

During JAK/STAT pathway activation and increased oxidative stress, many waste proteins are generated, as well as many irreversibly oxidant-damaged and potentially toxic proteins. All these require increased proteolytic degradation machinery for their degradation to preserve cell viability and basic cellular functions (22, 23). The constitutive proteasome may not be sufficient to accomplish this extra effort, and hence the immunoproteasomes are recruited and assembled, mainly induced by type 1 IFNs themselves.

### Proteasome Dysfunction and CANDLE Syndrome

In CANDLE syndrome (Figure 3B), proteasome system dysfunction leads to an inability of the cell to get rid of waste proteins. This situation may lead to a weak or moderate state of proinflammation in the absence of triggers, but under situations of stress, such as cold, viral infections, or physical stress, higher requirements of removing waste proteins are not met.

When a cell is infected by a virus, viral genetic material is sensed in the cytoplasm and activation of the central protein STING (stimulator of interferon genes) ensues. As a result, type 1 IFN genes are transcripted, and IFNs are released (24, 25). As stated above, when the IFN receptor is activated by IFNs, many waste proteins are produced, which must be removed by the proteasome and the IFN-induced immunoproteasome. On the other hand, cells infected by viruses produce viral proteins that are also a substrate for the proteasome system for their removal. Other triggers causing cellular stress also produce type 1 IFN release. If the proteasome system does not work properly, waste proteins accumulate in the cell and are also further marked with more ubiquitin (poly-ubiquitinization) (7, 8). The accumulation of poly-ubiquitinized proteins causes further cellular stress and more type 1 IFN production, thus feeding a vicious cycle of inflammation (2, 7–9).

Type 1 IFNs are virtually produced by every cell in the body, but the plasmacytoid dendritic cells are the most potent producers of type 1 IFNs and are involved in the pathophysiology of CANDLE (26). Type 1 IFNs exert many different actions (27). They increase release of proinflammatory substances and recruit inflammatory cells, including neutrophils and myeloid cells (28). Due to the enhanced and continuous type 1 IFN release, myeloid cells are rapidly mobilized from the bone marrow, and thus “atypical” or immature cells reach the target organs, contributing to the “atypical” skin infiltrate (26).

The IFN signature is very strong in CANDLE syndrome. Microarray analyses have shown an intense expression of IFN signature genes, and the serum of patients with CANDLE contains high levels of proteins of the IFN pathway such as IFN-derived protein 10 (2).

## Genetic Background

The first gene mutations detected in patients with CANDLE syndrome were located in the gene *PSMB8* (proteasome subunit, beta-type, 8) in chromosome 6p21.32, encoding for the β5i (i = inducible) subunit of the immunoproteasome (2, 6–8). Mutations in PSMB8 were responsible for CANDLE, JMP, and Nakajo–Nishimura syndromes. However, mutations in other genes, encoding other proteasome–immunoproteasome subunits or the regulatory protein *POMP*, were later discovered in patients with CANDLE syndrome, thus expanding the CANDLE genotype (9). All mutations were located in highly conserved sites in vertebrates and were thus predicted to be pathogenetic. It was proven that CANDLE syndrome is a disease of proteasome–immunoproteasome dysfunction, which could be inherited as a recessive homozygous, compound heterozygous or digenic trait, or less commonly in a dominant fashion (9). The following mutations have been so far identified in patients with CANDLE (9):

- *PSMB4 mutations*. The gene *PSMB4* (proteasome subunit, beta-type, 4), located in chromosome 1q21, encodes for the β7 subunit of the proteasome. It seems to be important for proteasome assembly and stabilization.
  The c.-9G>A mutation originates a β7 subunit protein with lower expression than that of wild type, which is therefore less incorporated into proteasome complexes than the wild-type counterpart. It is also possible that this mutant β7 subunit also impairs propeptide cleavage of the β5i subunit. A deletion of three aminoacids in β7, p.D212-V214, affects the N terminus of an α-helix forming an intramolecular hydrogen-bonding network that stabilizes its C-terminal extension. The C-terminal extension is essential for proteasome assembly (29). Two other mutations affect the C-terminal extension: (1) the c.44insG insertion, which causes a frameshift mutation (p.P16Sfs*45) and leads to non-expression of the mutant allele; and (2) the missense p.Y222X, which causes the loss of the C-terminal extension of the β7 subunit; although β7 subunit is expressed, it fails to incorporate into the 20S or 26S proteasome complexes.
  Finally, the deletion causing the p.D212_V214del mutant of β7 leads to a poor maturation of the β7 subunit. Although the mutant protein is detected in proteasome assembly intermediates, it is poorly incorporated into 20S or 26S proteasomes.
- *PSMA3 mutations*. The gene *PSMA3* (proteasome subunit, alpha-type, 3), located in chromosome 14q23.1, encodes for the α7 subunit of the proteasome.
  Two mutations in *PSMA3* have been reported in patients with CANDLE syndrome. A p.R233del deletion (c. 696_698delAAG) most likely affects the subunit folding and prevents incorporation of the subunit to the mature proteasomes. Overall reduced proteasome content is thus resulting (9). On the other hand, a c.404+2T>C mutation affects a splice site and causes an unstable transcript due to exon 5 skipping.
- *PSMB8 mutations*. The gene *PSMB8* (proteasome subunit, beta-type, 8), located in chromosome 6p21.32, encodes for the β5i subunit of the immunoproteasome. Incorporation of β5i subunit to the maturing immunoproteasome requires proteolytic removal of a prosequence by proteolytically active subunits. The β5i subunit has chemotrypsin-like activity, crucial for the immunoproteasome function.
  Mutations in PSMB8 in CANDLE syndrome may affect the chemotrypsin activity or impair immunoproteasome assembly or maturation. The most common mutation in CANDLE syndrome is T75M; when found in homozygosis, it causes selective impairment in chemotryptic-like activity. The A92T mutation produces a similar effect, as well as the mutations K105Q and M117V.
  The K105Q mutation is also associated with defects in incorporation and/or maturation of proteasome subunits and with inability to completely trim the β5i propeptide (9). The common T75M and the G201V mutations also cause decreased proteasome assembly (7, 8). Finally, the C135X mutation leads to truncation and non-expression of the protein (2, 9); when found in homozygosis, the subunit β5i is absent in all immunoproteasomes and most likely impairs immunoproteasome assembly, thus showing reduction in all three proteasome activities (trypsin-like, caspase-like, and chemotrypsin-like) (9).
- *PSMB9 mutations*. The gene *PSMB9* (proteasome subunit, beta-type, 3), located in chromosome 6p21.32, encodes for the β1i subunit of the immunoproteasome. The β1i subunit has a caspase-like proteolytic activity.
  The only β1i variant described so far is a missense mutation, p.G165D, located in a loop interconnecting 2 α-helices that define the position of a β1i/caspase-like activity conferred by threonine (9).
- *POMP mutations*. The gene *POMP*, located in chromosome 13q12.3, encodes for the POMP, which is key for the maturation and assembly of the proteasome subunits. POMP associates specifically with proteasome precursor intermediates and facilitates the sequential assembly of β subunits onto the preformed α subunit rings (15).
  A single patient with CANDLE has been found to bear no mutations in proteasome subunit genes, but a heterozygous, dominant, insertion in POMP causing a frameshift, p.E115Dfs*20 (c.344_345insTTTGA) and a truncated protein, which is likely unstable. POMP insufficiency causes proteasome precursor accumulation, reduced mature proteasome formation, and reduced overall proteasome activity (9).

Patients with CANDLE have shown variable combinations of these mutations (9). Most frequently, patients are homozygous or compound heterozygous for *PSMB8* mutations, but others are compound heterozygous for *PSMB4*, or are heterozygous for combinations such as *PSMA3*/*PSMB8, PSMB9*/*PSMB4*, or *PSMB8*/*PSMB4*. In the latter situation, a digenic inheritance is suggested causing additive proteasome defects. Patients with digenic inheritance have variable proteolytic defects. For example, a combination of *PSMB8*/*PSMA3* causes impairment in all three proteolytic activities, somewhat similar to patients’ compound heterozygous for *PSMB4* in whom proteasome assembly is severely impaired. Patients with combined *PSMB9*/*PSMB4* mutations have reduced caspase-like activity, which is conferred by subunit β1i. Finally, patients with double *PSMB8* mutations experience a severe decrease in chemotrypsin-like activity.

## Clinical Features

### Onset and Course

CANDLE syndrome usually starts in the first months of life (1, 30). The most common presenting sign is fever or temperature elevations below 38.3°C. These appear daily or almost daily, but the general state is minimally affected or even normal. Sometimes, cold exposure can trigger temperature elevation and skin lesions. Skin lesions are the first clinical sign to appear in CANDLE, and usually are present all along the disease course, although they may be less conspicuous after puberty. Lipodystrophy usually starts in early childhood and is usually well established before puberty. Finally, disabling joint manifestations usually occur in the long term. During patients’ life, different acute attacks of disease may ensue, spontaneously or after common triggers, which may affect virtually every organ in the body.

### Skin Manifestations of CANDLE

**Figure 4:**
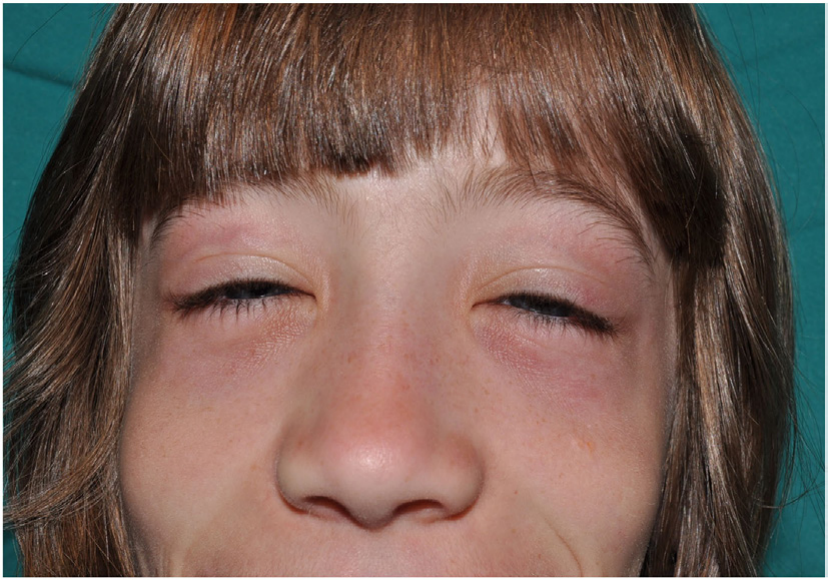
Periorbital erythema and edema and flat nose in a patient with CANDLE.

**Figure 5:**
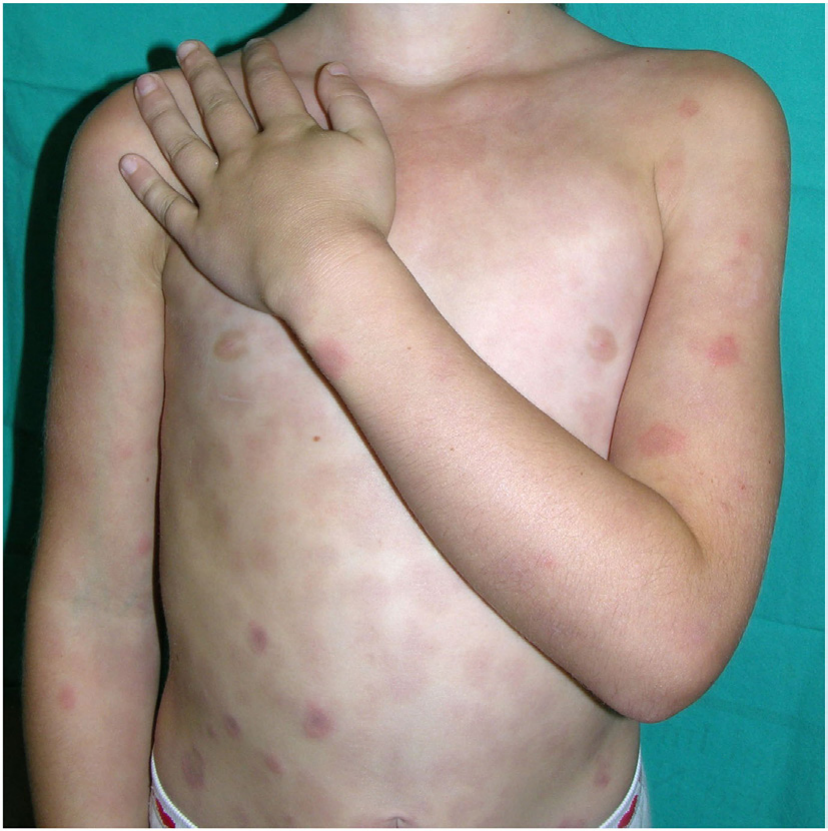
Annular, purpuric plaques of CANDLE syndrome.

**Figure 6:**
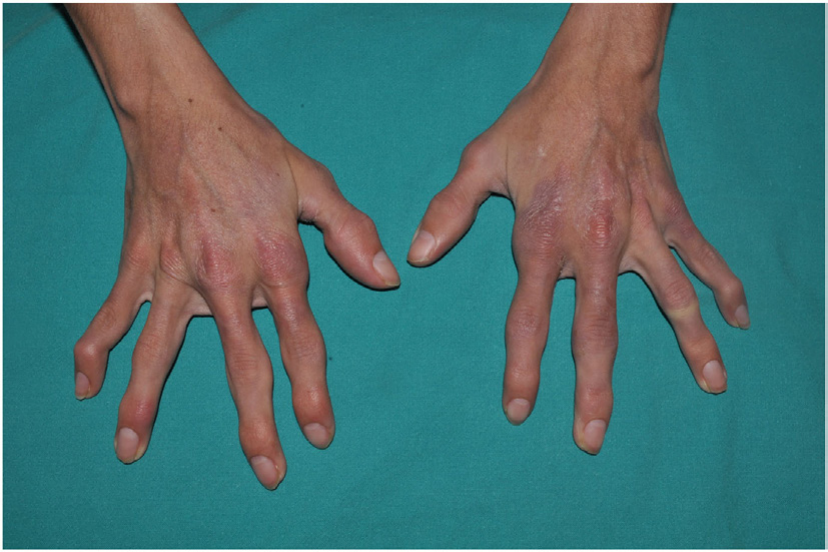
CANDLE hands: skin lesions and swollen joints.

The skin lesions of CANDLE syndrome are very characteristic and should raise the diagnosis. The combination of fever, typical skin lesions, and classic histopathologic features should allow for a rapid diagnosis of CANDLE (Figures 4–6). The skin lesions in CANDLE are of three types (1):

1. Acral, perniotic lesions. These usually appear in newborns and infants and are not regularly seen in childhood or later. They consist of intense, red or purplish, edematous plaques mostly located on the nose, ears, fingers, or toes. Cold may be a trigger for these lesions, but often there is no history of cold exposure.
2. Annular plaques. These lesions usually start in infancy or childhood and consist of erythematous or purpuric edematous lesions, often with annular shape with raised borders and a flat, purpuric center. They may appear in crops or individually and tend to fade within days or weeks, leaving a purpuric macule. New, active lesions coexist all over time with residual, purpuric macules, which confers a very typical appearance to the patients. These lesions are very conspicuous during childhood, but in adult life they may be less visible and may be absent in long-standing disease.
3. Perioral and periocular edema. Patients with CANDLE develop in infancy or childhood a persistent erythematous to violaceous edema affecting the periorbital and less commonly the perioral area. It may be less visible after puberty and in long-standing disease.

The histological features of the skin lesions in CANDLE are very characteristic and may permit a diagnosis in early stages of the disease (1, 27). The papillary and reticular dermis contains a perivascular and interstitial infiltrate of varying intensity, extending to the subcutaneous fat as lobar panniculitis. The infiltrate is predominantly composed of mononuclear cells, many of which have large, irregularly shaped nuclei; the atypical appearance of the infiltrate may lead to a diagnosis of skin malignancy. The infiltrate also contains mature neutrophils, some eosinophils, and a few mature lymphocytes. Leukocytoclasia is often seen, without fibrinoid necrosis of the vessels (1, 27). Immunohistochemistry shows that the infiltrate is mixed, with an important presence of myeloid cells (positive for myeloperoxidase) and a prominent cell population of macrophages (positive for CD163 and CD68/PMG1) (27). CD123-positive plasmacytoid dendritic cells are seen in clusters (27).

### Lipodystrophy

Fat loss is a key manifestation of CANDLE (1, 3, 30–32). It can be seen in most patients before the age of two, but it is progressive and may take some years to become fully developed. The loss of subcutaneous fat usually starts on the face and progresses to the trunk and upper limbs. The lower limbs are usually less affected. The cause of lipodystrophy is not well known, but it can be related to chronic inflammation involving the fatty tissue (33, 34). Alternatively, increased expression of proinflammatory cytokines in adipose tissue and reduced secretion of adiponectin and leptin may be involved (35, 36). An intense type 1 IFN signature is believed to be associated with fat loss in children with lipoatrophic panniculitis (37), which reinforces the role of IFN in CANDLE syndrome. Type 1 IFNs may be toxic to adipocytes, as is suggested by the development of lobar panniculitis with lipophagia and lipoatrophy in patients treated with intramuscular injections of IFN-β (38, 39).

Lipodystrophy and the typical skin lesions confer to CANDLE patients a unique phenotype. On the face, the loss of fat on the cheeks and the periorbital and periocular edemas are pathognomonic (1). In adulthood, the eyelids and the lips are retracted, causing a false proptosis and exposure of the teeth; coupled with the severe fat loss, these features cause an unmistakable appearance (4). On the limbs, a progressive fat loss, coupled with muscle wasting (see later), is seen. A prominent abdomen may be related to increased visceral fat, which remains the only fat storage capability of the patient. An increased distance between nipples is also typical in CANDLE (1). Metabolic disturbance due to absence of body fat can also impair hydrocarbon metabolism and lead to acanthosis nigricans and hirsutism.

### General Examination

Patients with CANDLE have a mild to moderate growth delay and show low weight and height (1, 30–32). Chronic inflammation may explain this physical delay, but muscle wasting and lipoatrophy are also major contributors. However, most patents with CANDLE do not show mental retardation.

CANDLE patients show variable degrees of hepatomegaly, which can be related to a secondary metabolic disturbance due to extensive lipoatrophy. Splenomegaly and generalized lymphadenopathy are common findings, reflecting the persistent autoinflammatory activity.

### Musculoskeletal Signs

Arthralgias are very common in children with CANDLE, but patients do not show radiologic features of arthritis. Some joint swelling can appear in the interphalangeal joints, but the absence of overt arthritis distinguishes CANDLE from rheumatoid arthritis or juvenile idiopathic arthritis (1, 30–32). With time, hyperextensibility of interphalangeal joints can occur, and during adulthood, most patients will develop variable degrees of joint contractures on the hands and feet, which are often severely disabling (4).

Cartilage inflammation has been reported in CANDLE patients (1). Recurrent and also chronic chondritis of the ears and nose causes partial loss of auricles and a flat, saddle-like nose. Because both ears and nose are usually exposed, a role of triggering by cold has been suspected.

Muscle involvement is also a feature of CANDLE (4). Acute attacks of inflammatory myositis have been reported that can be demonstrated by MRI. Possibly, there is also a role for chronic muscle inflammation in the development of severe muscular wasting.

### Nervous System

As stated above, mental delay is not a common feature in CANDLE (1). However, patients may suffer attacks of aseptic meningitis, meningoencephalitis, and possibly some degree of chronic inflammation in the CNS. Basal ganglia calcifications have been reported (1), most likely as a result of encephalitis; these are similar to those present in Aicardi–Goutières syndrome, which also features a high type 1 IFN production.

### Other Organ Involvement

Episodes of inflammation may occur in any organ, as well as some degree of persistent generalized inflammation. Attacks of acute sterile epididymitis, conjunctivitis and nodular episcleritis, parotitis, pneumonitis, nephritis, carditis, and otitis have been reported. The clinical manifestations are related to the organs involved. Some of these complications have been reported to be fatal (1).

## Laboratory Investigations

As is the case with other AIDs, laboratory findings are not very striking (1, 3, 32). The most common features are elevation of acute phase reactants (ESR, CRP, and thrombocytosis) and a chronic, hypochromic anemia. Liver enzymes are usually moderately elevated, but this may be caused by lipodystrophy itself; also, increased triglyceride levels can occur in relation to metabolic disturbance by lipodystrophy. Less frequently, elevated muscle enzymes (CPK and aldolase) reveal chronic muscle involvement (1). Studies for autoimmunity and autoantibodies are usually negative, but some patients show increased levels of antinuclear antibodies. Serum levels of immunoglobulins are regularly normal. Bone marrow and lymph node biopsies have been unconspicuous, revealing only reactive changes.

Other laboratory and imaging anomalies are seen during acute inflammatory attacks; these are dependent on the organs affected by inflammation.

## Diagnosis and Differential Diagnosis

The diagnosis of CANDLE is suspected by the early onset of fevers, skin lesions, and lipodystrophy. A skin biopsy with immunohistochemistry studies can be characteristic enough to permit an accurate diagnosis. The genetic study of the genes involved establishes a confirmation diagnosis.

Other AIDs may share some features with CANDLE syndrome, including NOMID syndrome, TRAPS, or hyper-IgD syndrome. Lipodystrophy in CANDLE syndrome is a very characteristic feature of the disease, but other causes of loss of fat must be considered, including generalized congenital lipodystrophy, partial familial lipodystrophy, leprechaunism, or acquired partial lipodystrophy of Barraquer–Simons. Aicardi–Goutières syndrome and other type 1 interferonopathies (such as SAVI syndrome, familial chilblain lupus, or C1q deficiency) may show features similar to CANDLE syndrome (28, 40, 41). Sweet syndrome in infants may present with violaceous ring lesions reminiscent of CANDLE syndrome, and histology may be misleading in some cases. Fat loss and skin lesions are clinical manifestations of a recently described autoinflammatory syndrome named otulipenia, due to loss-of-function mutations in OTULIN, encoding a deubiquitinase that cleaves Met1-linked chains (42).

## Prognosis and Follow-Up

CANDLE patients have a variable outcome. Some patients have had a lethal course due to acute attacks of inflammation in important organs of the body. In other patients, a long survival is possible, with variable degrees of disability (1, 3, 4).

Regular clinical follow-up is mandatory. A protocol has not been established, but attention must be paid to identify inflammatory attacks as early as possible. Regular skin, eye, and joint exams are recommended. Endocrinologist consultation is mandatory for diet and metabolic control because of lipodystrophy. Basic laboratory follow-up must include CBC (with special attention to anemia, leukocytosis with increased neutrophil count, and thrombocytosis), ESR, CRP, serum liver enzymes, muscle enzymes, and metabolic profile (including glucose, triglyceride, and cholesterol levels). Ultrasound may be helpful in detecting enlarged liver, spleen, or lymph nodes. Specific tests for organ involvement must be considered in patients with abnormal clinical examination.

## Treatment

So far, no individual treatment has been consistently effective in CANDLE syndrome. Oral corticosteroids and methotrexate can provide some improvement. Methotrexate can be considered the first line therapy. NSAIDs may provide partial control of fevers. Dapsone or colchicine has been ineffective. Cyclosporine, azathioprine, or intravenous immunoglobulins have achieved minimal improvements, if any. Anti-TNF drugs such as etanercept have not been helpful and have even been the cause of disease exacerbations (1). Acute attacks may need systemic corticosteroids as well as organ-specific therapy.

A compassionate use treatment protocol has been started for CANDLE syndrome with the selective JAK1/2 kinase inhibitor baricitinib. Oral baricitinib was used in patients who failed to achieve control or required high doses of corticosteroids. Eight patients treated with this drug showed clinical and analytical improvement (43), but these results still await confirmation.

Finally, physical therapy to prevent joint contractures and specific organ therapy must be provided.

## Concluding Remarks

CANDLE syndrome is an AID due to gene mutations leading to protesome–immunoproteasome dysfunction. CANDLE syndrome can be diagnosed very early in life because the skin signs and their histopathology are very characteristic. Genetic confirmation is necessary. Early therapy to prevent disabling manifestations is desirable, but still no agent has been truly effective. Prevention and treatment of acute inflammatory attacks will permit longer life expectancy in these patients.

## Author Contributions

There is one single author for this article. The manuscript has been completely written by AT.

## Conflict of Interest Statement

The author declares that the research was conducted in the absence of any commercial or financial relationships that could be construed as a potential conflict of interest.
